# ﻿A new species of *Siphlonurus* Eaton, 1868 (Ephemeroptera, Siphlonuridae) from Yunnan, China

**DOI:** 10.3897/zookeys.1166.102847

**Published:** 2023-06-08

**Authors:** Kun Yang, Xian-Fu Li, Xiao-Li Tong, Qing-Hua Cai

**Affiliations:** 1 Institute of Eastern-Himalaya Biodiversity Research, Dali University, Dali 671000, Yunnan, China; 2 Collaborative Innovation Center for Biodiversity and Conservation in the Tree Parallel Rivers Region of China, Dali University, Dali, Yunnan, China; 3 Research Center of Ecology and Governance for Er’hai Lake Streams, Dali, Yunnan, China; 4 Department of Entomology, College of Plant Protection, South China Agricultural University, Guangzhou 510642, Guangdong, China; 5 State Key Laboratory of Freshwater Ecology and Biotechnology, Institute of Hydrobiology, Chinese Academy of Sciences, Wuhan 430072, Hubei China

**Keywords:** Hengduan Mountains, Himalaya, Mayfly, Siphlonuridae

## Abstract

*Siphlonurusdongxi* Li & Tong, **sp. nov.** from Shangri-La City, Yunnan Province, China, is described based on egg, nymph, and winged stages. The new species is closely related to *S.davidi* (Navás, 1932), and can be distinguished by the colour of the imago, the forking point of MP, the penis, posterolateral spines of tergum IX of imagoes, and first abdominal terga nymph, as well as the structure of the egg. The new species and *S.davidi* have the same morphological and structural characteristics, such as the long cubital area with many intercalaries, cross veins between C, Sc, RA, and RSa_1_ surrounded with distinct pigments, the strong curvature of vein CuP in the forewing, the broad expansion of the hindwing, the membranous penis lobes fused without teeth, supporting the proposition of a new species complex, the *Siphlonurusdavidi* group. The structures of the penis and the egg of the new species could help understand the origin and evolution of the genus *Siphlonurus*.

## ﻿Introduction

The genus *Siphlonurus* Eaton, 1868 (Ephemeroptera, Siphlonuridae) is characterized by many plesiomorphies (Kluge et al. 1995). About 40 *Siphlonurus* species have been reported from the Nearctic and Palaearctic realms (Kluge 2004). A few species have been mentioned from China, but Kluge (2004) suggested that there was an “unknown group” of *Siphlonurus* in China. So far, only *S.davidi* (Navás, 1932) is distributed in China. It was initially described from a single male subimago (Zhou and Peters 2003), while the type specimen was redescribed later (Sartori and Peters 2004). Afterwards, the egg, nymph, and imago of *S.davidi* were described by Han et al. (2016). Consequently, the lack of research on adults of *Siphlonurus* might limit the understanding of this genus in China.

*Siphlonurusdavidi* presents some plesiomorphies, indicating a close relationship with the ancestor of the *Siphlonurus* lineage, such as the forking point of MP subequal to that of the fusion point of MA and RS, the cubital area longer and with more intercalaries between CuA and the posterior margin of the wing, and the hindwings approximately half the length of the forewings, longer than in other *Siphlonurus* species (Sartori and Peters 2004; Han et al. 2016). During our recent survey of the mayfly fauna of the Hengduan Mountains area, at the eastern end of the Himalayas, a not yet described species of *Siphlonurus* similar to *S.davidi* was found in Shangri-La City, western Yunnan, China, at an altitude of more than 3000 m. Here, we describe this new *Siphlonurus* species based on imago, subimago, nymph, and egg stages.

## ﻿Materials and methods

*Siphlonurus* nymphs were collected with a D-frame net from the floodplain habitats of the Dugang River in Shangri-La City, northwestern Yunnan, China. Following the guidelines from Li et al. (2022), the habitat photographs were taken using a Huawei Nova 8 mobile phone equipped with a Kase 40–75 mm macro lens. Some specimens were dissected under a stereomicroscope and were mounted on slides with Hoyer’s solution for examination with a digital microscope. Slide-mounted specimens were examined and photographed with a Keyence VHX-S550E digital microscope. For scanning electron microscopy (SEM), eggs were dried, coated with gold, and observed with a VEGA3 SBU SEM (Tescan, Brno, Czech Republic). Measurements were taken using ImageJ image processing software. The final plates were prepared with Adobe Photoshop CC 2018.

All examined materials were deposited at the Museum of Biology, Institute of Eastern-Himalaya Biodiversity Research, Dali University, Dali, Yunnan, China (MBDU).

## ﻿Results

### ﻿ Siphlonurus dongxi

Taxon classificationAnimaliaEphemeropteraSiphlonuridae

Li & Tong sp. nov.

7C6AEA5D-558A-5874-B7C1-D4001161F3C7

https://zoobank.org/27AA2276-1CF5-4CA9-A6ED-5FCC5EC9F691

Figs 1 , 2 , 3 , 4 , 5 , 6 , 7 , 8 , 9 , 10 , 11 , 12 , 13 , 14 , 15 , 16 , 17

#### Material examined.

***Holotype***: male imago, with final nymphal instar exuvia (in ethanol), China, Yunnan Province, Shangri-La City, Jiantang Town, Dugang river, 27°47′50.4″N, 99°48′43.3″E, 3361 m a.s.l., 12.VI.2022, coll. Xian-Fu Li. ***Paratypes***: 13 nymphs, 30 imagoes and 8 subimagoes reared from nymphs with same data as holotype. 5 nymphs from same location as holotype, but 1.VI.2021, coll. Yi-Hao Fang.

#### Diagnoses.

The new species is similar to *S.davidi*. It can be distinguished from *S.davidi* by the colour of the imago, the morphological structure of egg, the forking point of MP, the transversal sclerite of the penis with two dorsal elongations, the dorsal elongation of the penis basally expanded, the elongations of the ventral sclerite, the posterolateral spines of tergum IX of imagoes and the first abdominal terga of the nymph, as well as the structure of the egg.

#### Descriptions.

***Male imago*** (in ethanol). Body length 18.4–20.5 mm (excluding cerci), head width 3.3–3.5 mm, forewing length 17.3–18.6 mm, hindwing length 7.8–8.1 mm, antennae 1.4–1.6 mm. Ratio of hindwing: forewing length about 0.43.

**Figure 1.**
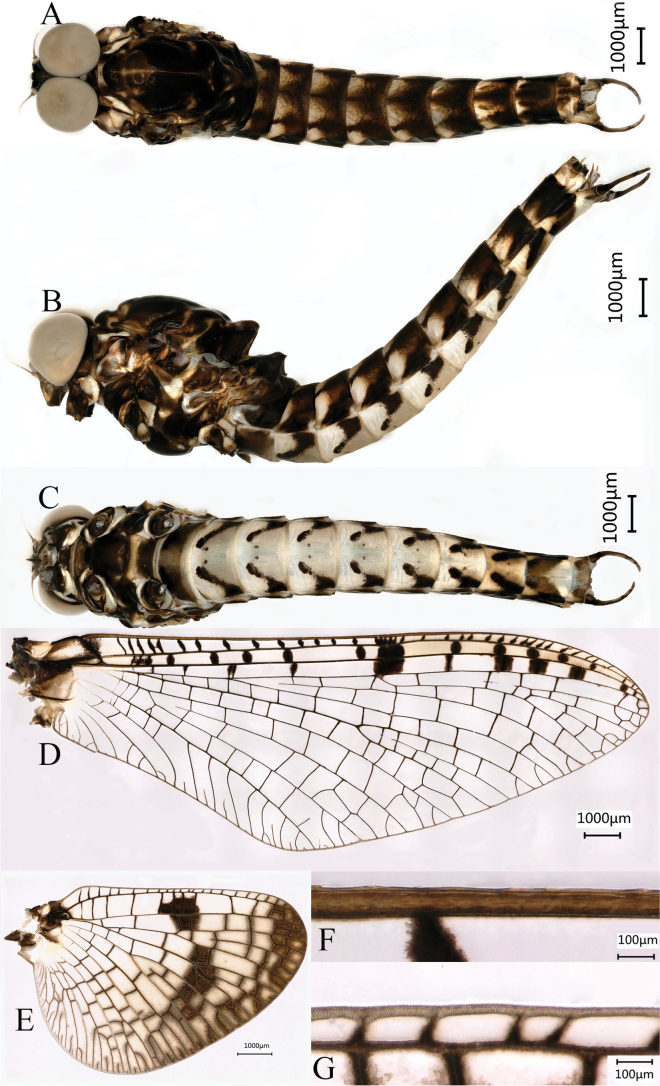
Male imago of *Siphlonurusdongxi* Li & Tong, sp. nov. **A** dorsal view **B** lateral view **C** ventral view **D** forewing **E** hindwing **F** anterior margin of forewing **G** anterior margin of hindwing.

***Head***: compound eyes contiguous (Fig. 1A), each of them spherical, upper portion and lower portion grey, without clear line between them (Fig. 1B).

**Figure 2.**
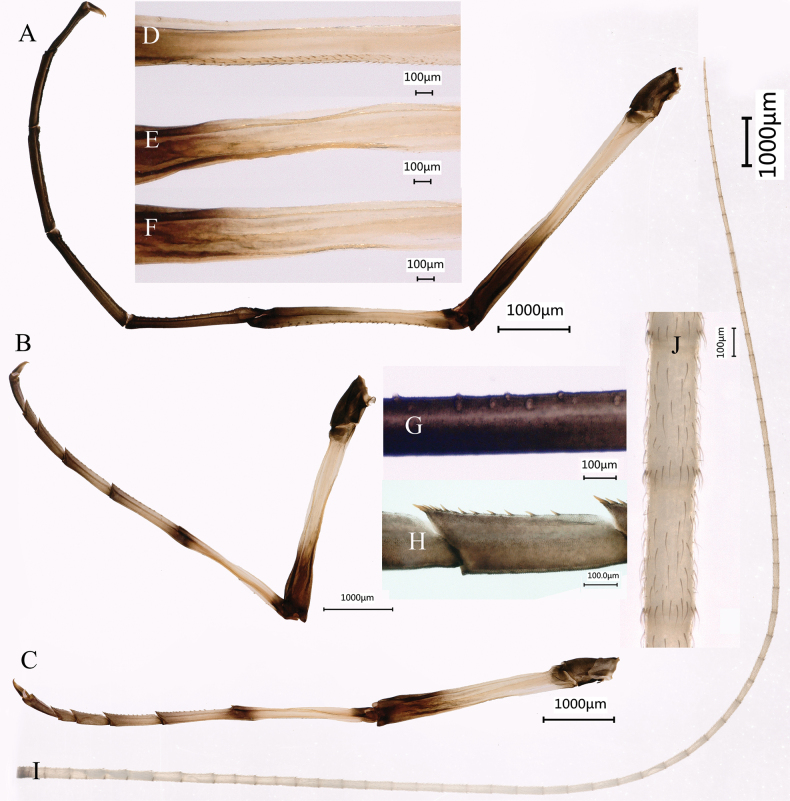
Male imago of *Siphlonurusdongxi* Li & Tong, sp. nov. **A** foreleg **B** midleg **C** hindleg **D** femur enlarged of foreleg **E** femur enlarged of midleg **F** femur enlarged of hindleg **G** tarsus enlarged of foreleg **H** tarsus enlarged of midleg **I** cerci **J** cerci enlarged.

***Thorax***: generally light yellow and dark brown, mesonotum anterior and legs basal with light yellowish stripes or rings (Figs 1A–C, 2A–C). All legs light yellowish to dark brown, with distinct markings at both ends of femur and tibia (Fig. 2A–C). Length of femur: tibia: tarsus of foreleg = 1.3: 1.0: 2.4, tarsal segments from basal to apical = 2.2: 2.0: 1.8: 1.4: 1.0; femur: tibia: tarsus of midleg = 1.4: 1: 1.6, tarsal segments from basal to apical = 3.7: 2.5: 1.8: 1.0: 1.4; femur: tibia: tarsus of hindleg = 1.5: 1.0: 1.6, tarsal segments from basal to apical = 4.3: 2.8: 1.9: 1.0: 1.8. Inner margin of foreleg tarsus densely covered with circular bulges (Fig. 2A, G), and that of midleg and hindleg with stout setae (Fig. 2B, C, H). Outer margin of foreleg femur and tibia relatively densely covered with stout setae (Fig. 2A–F). Similar to other *Siphlonurus* species, all legs end with one blunt and one hooked claw. Forewings (Fig. 1D) generally hyaline except outer 1/3 of C and Sc, Sc and RA fields, base of forewing distinctly pigmented with black inlaid with yellow. Cross veins between C, Sc, RA, and RSa_1_ surrounded with distinct pigments, with dark spots. The forking point of MP is more proximal from that of fusion point of MA and RS. Anterior margin of forewing with small setae (Fig. 1F). Base of hindwing (Fig. 1E) distinctly pigmented with black inlaid with yellow, an additional large dark patch in the middle of Sc and RA cells, RA, and RS cells. Distal half of hindwing washed with black, making this area semitransparent, areas near centre and near margin of hindwing darker than others. Ratio of width: length about 0.79. Anterior margin without setae (Fig. 1G).

**Figure 3.**
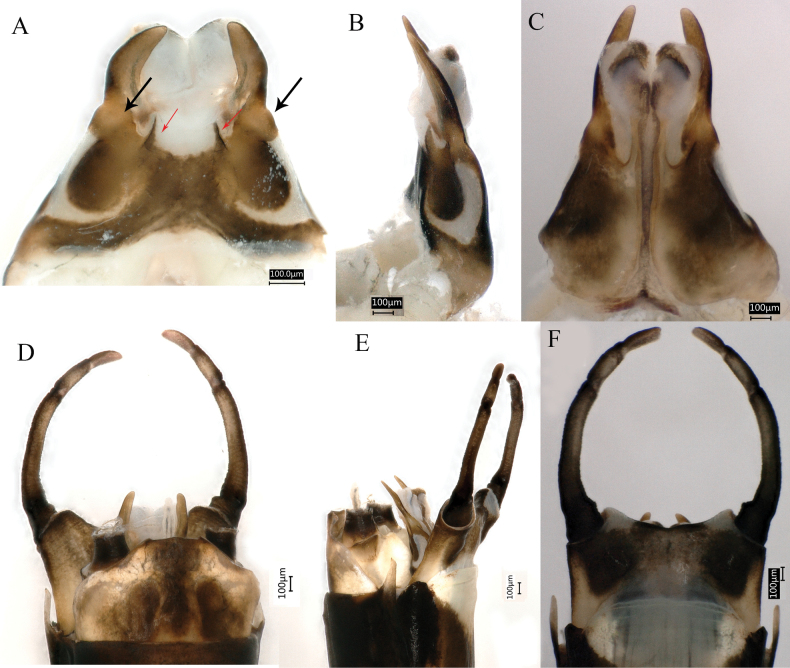
Male imago of *Siphlonurusdongxi* Li & Tong, sp. nov. **A** dorsal view of penis **B** lateral view of penis **C** ventral view of penis **D** dorsal view of genitalia **E** lateral view of genitalia **F** ventral view of genitalia.

***Abdomen*** (Fig. 1A–C) light yellow and dark brown, dorsal (Fig. 1A) and lateral sides (Fig. 1B) of terga II–X each with “W” shaped dark stripes; the ventral side (Fig. 1C) of terga II–VIII each with two dark symmetric spots and dark stripes, part and all of terga VIII–X distinct pigmented. Posterolateral spines of tergum IX well developed (Fig. 3D–F). Middle filament lost, terminal filament vestigial. Cerci lengths 29.5 mm, densely covered with long setae (Fig. 2I, J).

***Genitalia*.** Penis relatively long (Fig. 3A–C), brown with dark brown markings, visible in distinct views (Fig. 3D–F); transversal sclerite with two elongations (Fig. 3A, indicated by red arrow) and two semicircular white plates shown in dorsal and lateral views (Fig. 3A, B); dorsal elongation of penis arched without spines, basally expanded (Fig. 3A, indicated by black arrow) apical half slim, penis lobe white fused without spines; elongations of ventral sclerite long, gradually widen from basal to subapical; styliger (Fig. 3D–F) 4-segmented, dark brown with stout setae, basal segment shortest but broadest, second segment about twice the length of third and apical ones together, the latter two subequal in length, each slightly longer than basal one. Styliger plate (Fig. 3F) slightly notched in middle.

**Figure 4.**
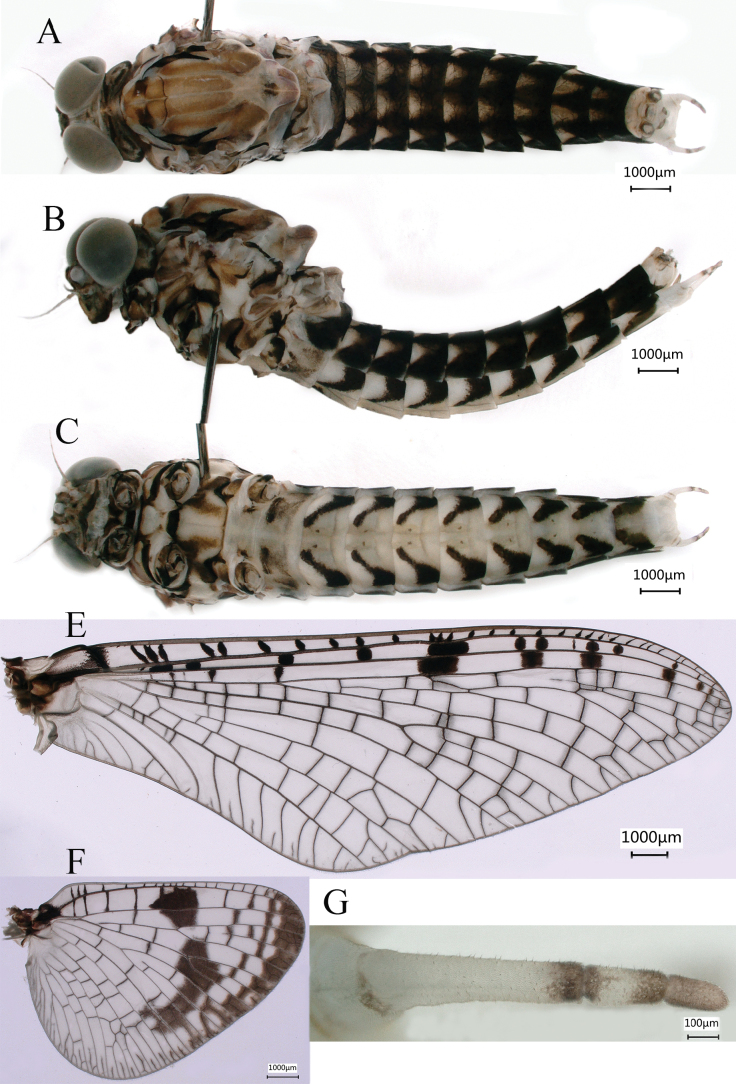
Male subimago of *Siphlonurusdongxi* Li & Tong, sp. nov. **A** dorsal view **B** lateral view **C** ventral view **D** forewing **E** hindwing **F** forceps of genitalia.

**Figure 5.**
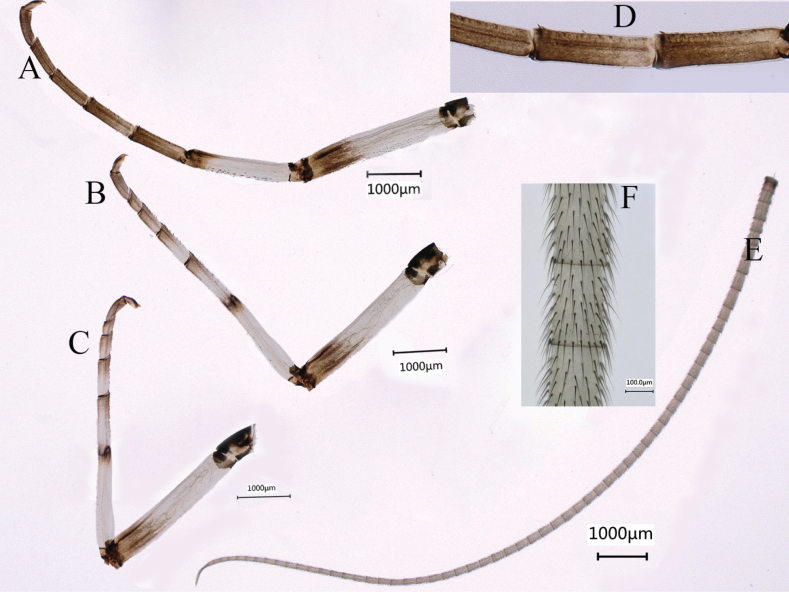
Male subimago of *Siphlonurusdongxi* Li & Tong, sp. nov. **A** foreleg **B** midleg **C** hindleg **D** tarsus enlarged of foreleg **E** cerci **F** cerci enlarged.

**Figure 6.**
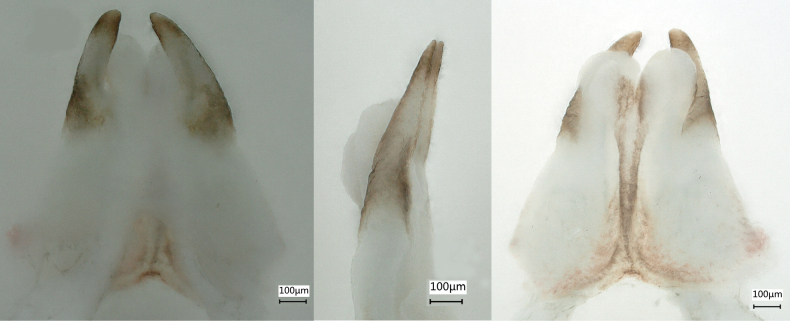
Male subimago of *Siphlonurusdongxi* Li & Tong, sp. nov. **A** dorsal view of penis **B** lateral view of penis **C** ventral view of penis.

***Male subimago*** (in ethanol) (Fig. 4A–G) similar to male imago except for following characters: thorax and terga VIII–X overall lighter than the male imago (Fig. 4A–C); forewing (Fig. 4E) and hindwing (Fig. 4F) subhyaline; forewing without intercalaries at MP_1_ to iMP, with tiny setae on outer and hind margins; length of femur: tibia: tarsus of foreleg (Fig. 5A) = 1.4:1.0: 2.0, tarsal segments from basal to apical = 1.8: 1.6: 1.3: 1.0: 1.0; femur: tibia: tarsus of midleg (Fig. 5B) = 1.5: 1.0: 1.7, tarsal segments from basal to apical = 3.2: 2.1: 1.4: 1.0: 1.4; femur: tibia: tarsus of hindleg (Fig. 5C) = 1.5: 1.0: 1.5, tarsal segments from basal to apical = 3.9: 2.3: 1.6: 1.0: 1.8; inner of tarsus of foreleg with circular bulges and setae (Fig. 5D). The sclerite structure of penis incomplete (Fig. 6A–C). Styliger plate only shallowly curved, posterior margin waved; styliger with relative densely thick setae (Fig. 4G). Forewing length 17.0–17.5 mm, hindwing length 7.8–8.1 mm, cerci length 15.4–16.5 mm (Fig. 5E, F).

**Figure 7.**
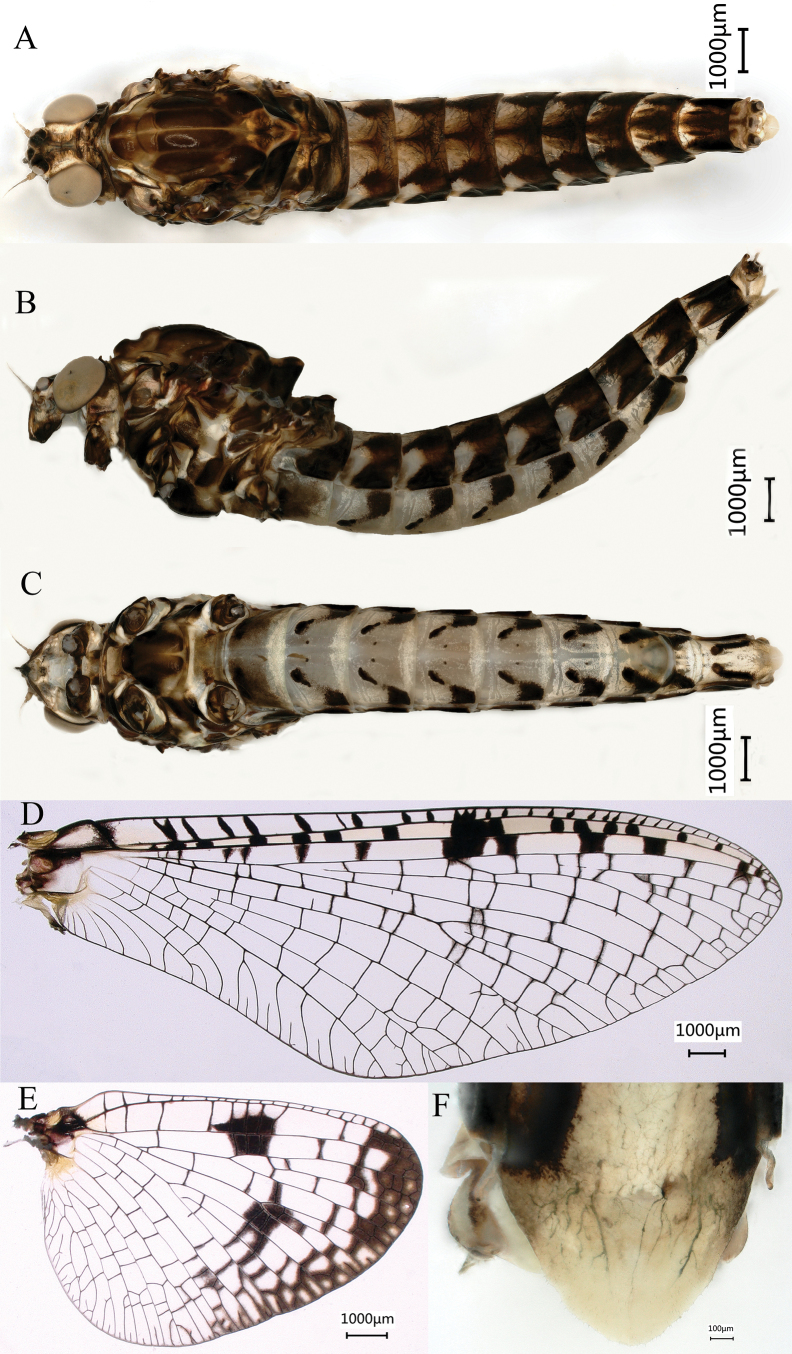
Female imago of *Siphlonurusdongxi* Li & Tong, sp. nov. **A** dorsal view **B** lateral view **C** ventral view **D** forewing **E** hindwing **F** posterior part of abdomen (ventral view).

**Figure 8.**
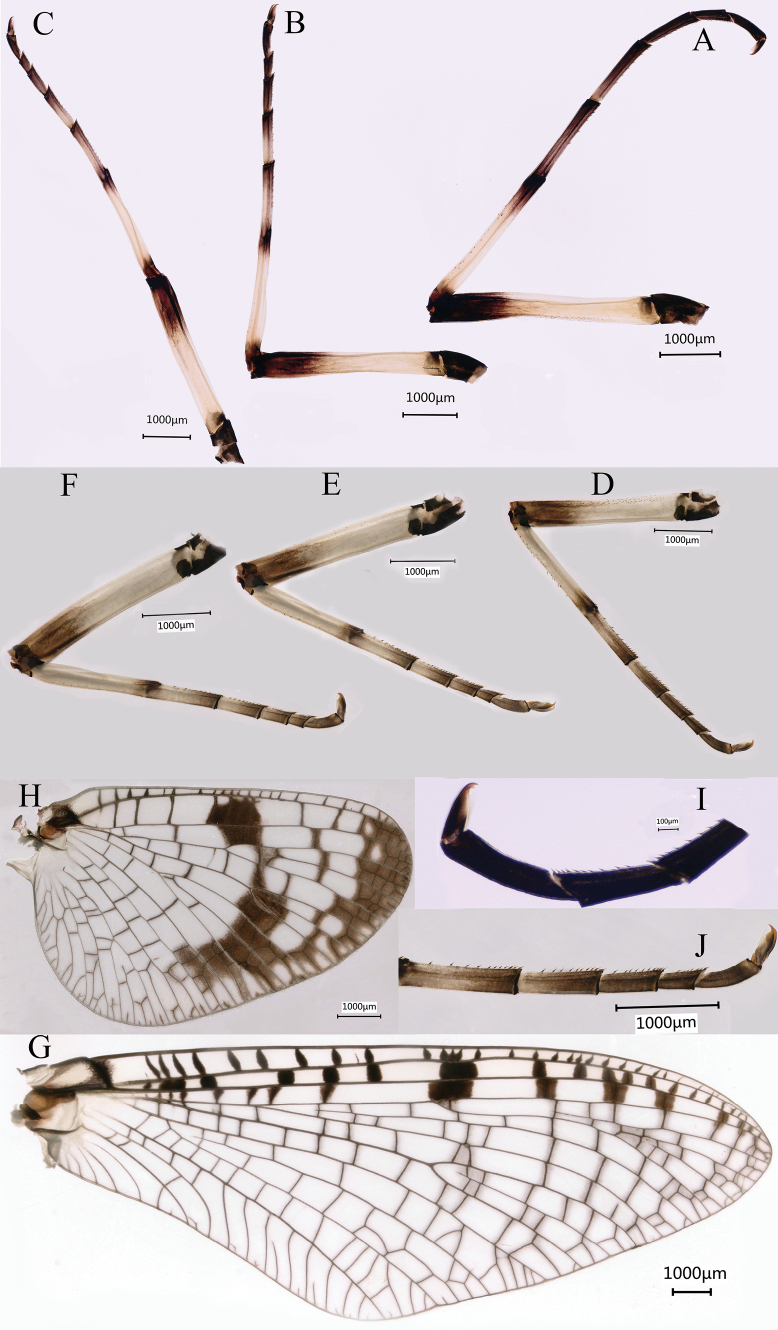
Female imago and subimago of *Siphlonurusdongxi* Li & Tong, sp. nov. **A** foreleg of imago **B** midleg of imago **C** hindleg of imago **D** foreleg of subimago **E** midleg of subimago **F** hindleg of subimago **G** forewing **H** hindwing **I** tarsus enlarged of imago **J** tarsus enlarged of subimago.

***Female imago*** (in ethanol). Colour pattern similar to male; body length 19.6–22.2 mm, head width 3.1–3.4 mm, caudal filaments 20.7–24.4 mm, forewing 18.5–21.3 mm, hindwing 9.0–9.4 mm (Fig. 7A–F). Length of femur: tibia: tarsus of foreleg = 1.3: 1.0: 1.8, tarsal segments from basal to apical = 3.0: 2.3: 1.7: 1: 1.3; femur: tibia: tarsus of midleg = 1.5: 1.0: 1.6, tarsal segments from basal to apical = 3.2: 2.3: 1.6: 1: 1.4; femur: tibia: tarsus of hindleg = 1.5: 1.0: 1.6, tarsal segments from basal to apical = 3.8: 2.8: 1.7: 1.0: 1.6. Subgenital plate produced to 1/3 length of sternum VIII (Fig. 7B). Compared with male, forewing of female imago with sparse cross veins between C and Sc; all of Sc and RS fields, and outer part of RA and RS cells subhyaline (Fig. 7D). Inner margins of tarsus of foreleg, midleg and hindleg densely covered with spines (Fig. 8A–C, I).

***Female subimago*** (in alcohol). Similar to male subimago except the tarsal segments of foreleg with more spines and usual sexual differences (Figs 8D–I). Length of femur: tibia: tarsus of foreleg = 1.4: 1.0: 1.6, tarsal segments from basal to apical = 3.1: 2.1: 1.5: 1: 1.6; femur: tibia: tarsus of midleg = 1.4: 1.0: 1.5, tarsal segments from basal to apical = 4.1: 2.6: 1.7: 1: 2.3; femur: tibia: tarsus of hindleg = 1.4: 1.0: 1.4, tarsal segments from basal to apical = 4.0: 2.5: 1.7: 1.0: 1.9. Inner margins of tarsus of foreleg, midleg and hindleg densely covered with spines (Fig. 8D–F, J). Forewing length 17.7–20.2 mm, hindwing length 8.9–9.1 mm, cerci length 13.2–14.2 mm.

**Figure 9.**
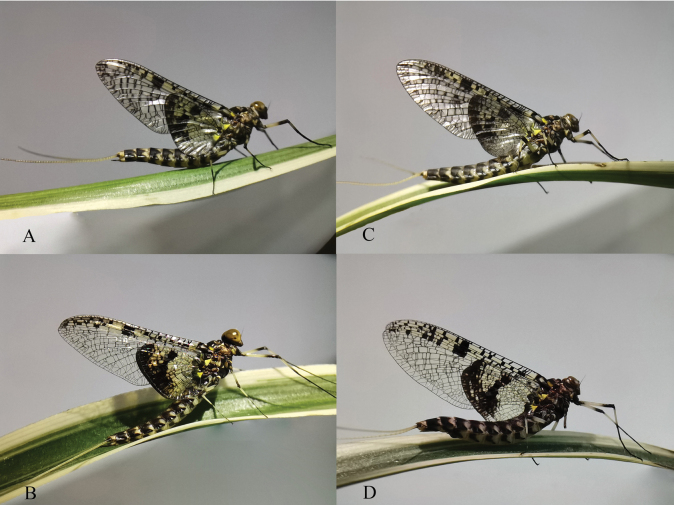
Winged stages of *Siphlonurusdongxi* Li & Tong, sp. nov (living) **A** male subimago **B** male imago **C** female subimago **D** female imago.

Winged stages of *Siphlonurusdongxi* Li & Tong, sp. nov. (living) are shown in Fig. 9A–D.

**Figure 10.**
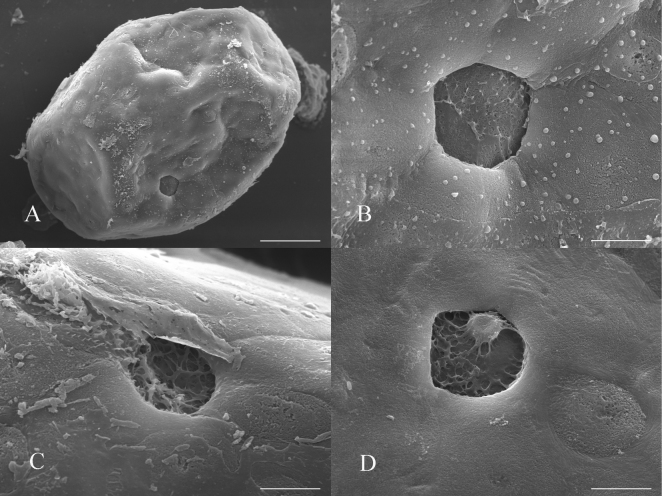
Egg of *Siphlonurusdongxi* Li & Tong, sp. nov. (SEM image) **A** shape and exochorionic surface of egg **B**–**D** micropyles enlarged. Scale bars: 50 μm (**A**); 10 um (**B–D**).

***Eggs.*** Oval with irregular flat areas (Fig. 10A), length of 224–240 um, and width of 168–175 um. Chorion without obvious reticulation, with micropyle with or without reticulation (Fig. 10A–D). No accessory attachment structure apparent, but the egg surface has convex rough structures (Fig. 10A, B). The eggs were stuck tightly together, and the mass remained intact when placed in the water or ethanol.

#### Persistent mouthparts of winged stages.

**Figure 11.**
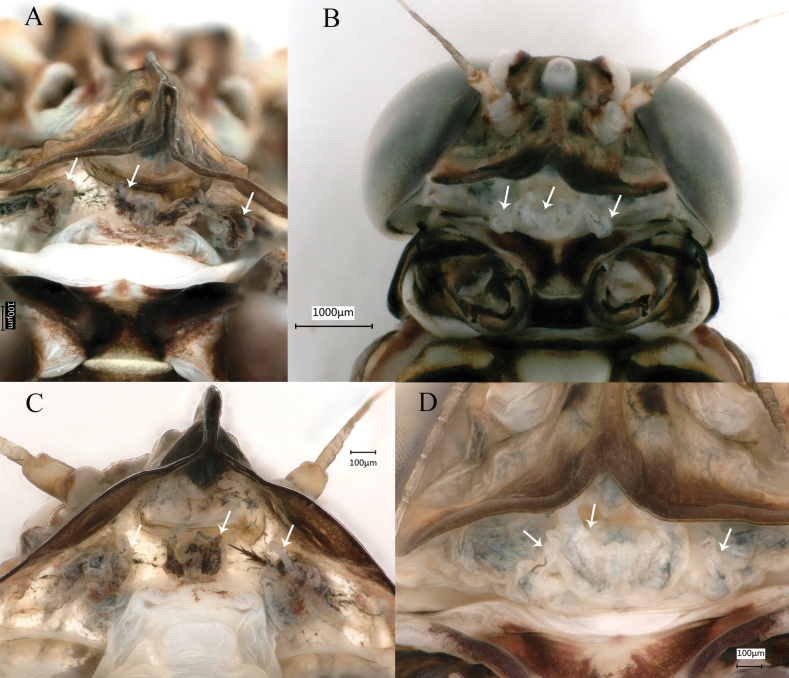
Persistent mouthparts of *Siphlonurusdongxi* Li & Tong, sp. nov. **A** male subimago **B** male imago **C** female subimago **D** female imago.

The new species presents persistent mouthparts in winged stages; in ventral view of head, the labial and maxillary are present and clearly visible (Fig. 11A–D, indicated by white arrow).

#### Final nymphal instar

**Figure 12.**
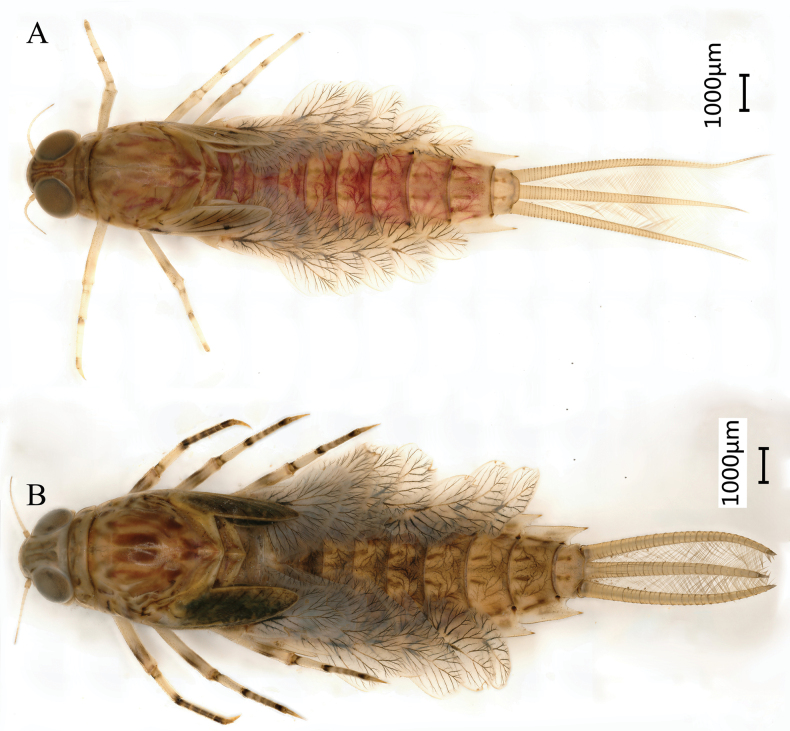
Last nymphal instar, dorsal habitus of *Siphlonurusdongxi* Li & Tong, sp. nov. **A** male (just after molt) **B** female.

**Figure 13.**
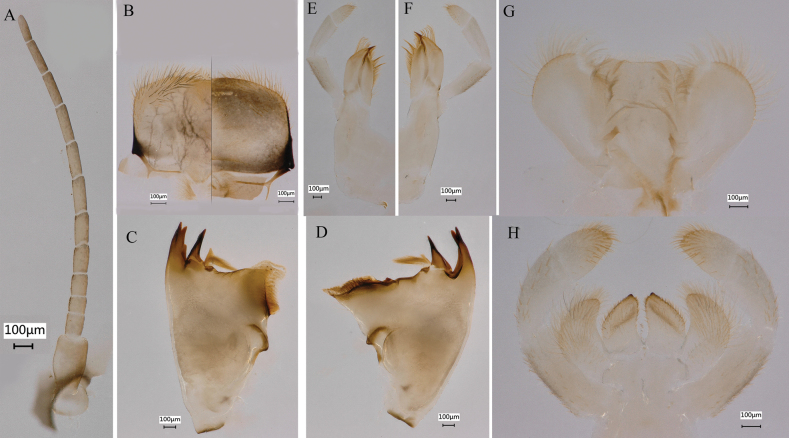
Nymphal characters of *Siphlonurusdongxi* Li & Tong, sp. nov. **A** antenna **B** labrum (ventral view on left, dorsal view on right) **C** left mandible **D** right mandible **E** left maxilla **F** right maxilla **G** hypopharynx **H** labium.

**Figure 14.**
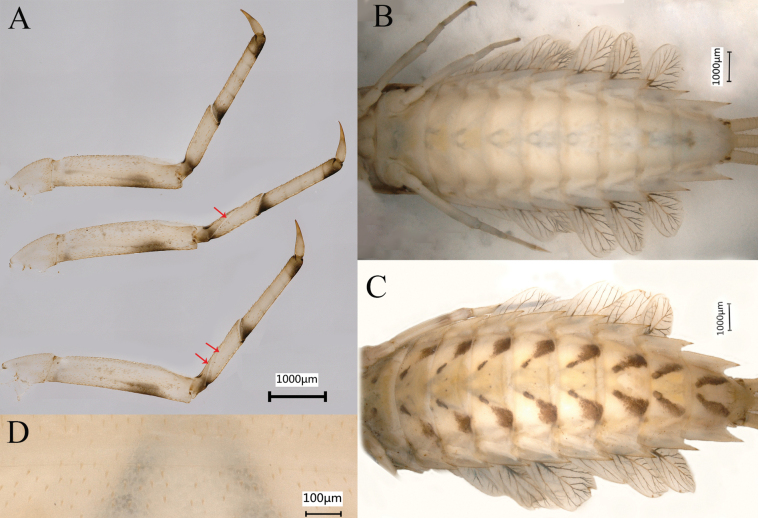
Nymphal characters of *Siphlonurusdongxi* Li & Tong, sp. nov. **A** legs (dorsal view), from top to bottom foreleg, midleg and hindleg **B** abdomen of the middle instar (ventral view) **C** abdomen of the last instar (ventral view) **D** enlarged of abdomen (ventral view).

**Figure 15.**
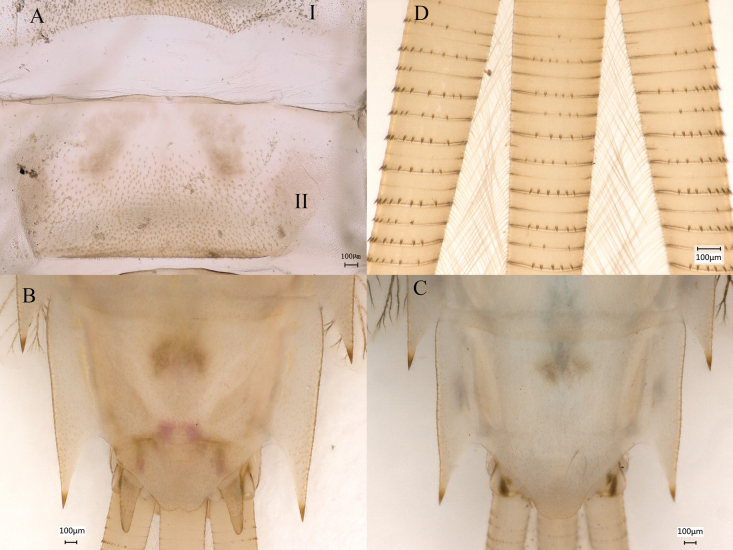
Nymphal characters *Siphlonurusdongxi* Li & Tong, sp. nov. **A** posterior part of abdomen (dorsal view) **B** posterior part of abdomen of male (ventral view) **C** posterior part of abdomen of female (ventral view) **D** enlarged of caudal filaments.

**Figure 16.**
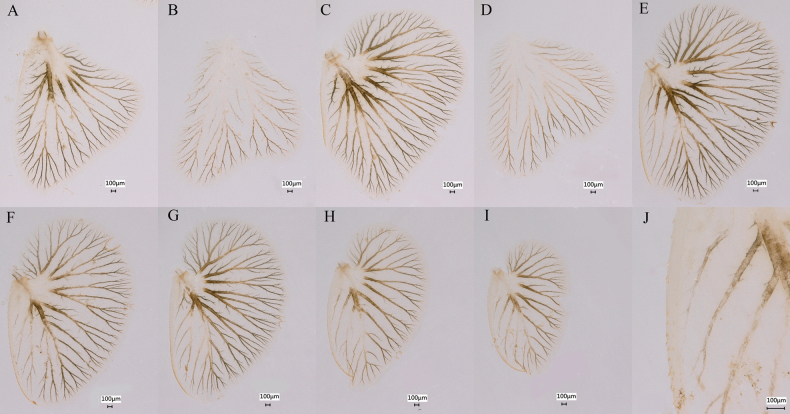
Gills of *Siphlonurusdongxi* Li & Tong, sp. nov. **A** dorsal lamella of gill I **B** ventral lamella of gill I **C** dorsal lamella of gill II **D** ventral lamella of gill **E** gill III **F** gill IV **G** gill V **H** gill VI **I** gill VII **J** anterior enlarged of gill VII.

(in ethanol) light yellow with red and dark markings (Fig. 12A, B), body length 14.1–16.7 mm (excluding cerci); head width 2.6–2.8 mm, cerci lengths 6.7–7.7 mm, median filament 5.6–6.8 mm, antennae 1.8–2.1 mm. Morphology and structure of the head (Fig. 12A, B) and mouthparts (Fig. 13A–H) of the new species similar to that of *S.davidi*. All legs similar (Fig. 14A), surface with short thick sparse setae, the apex of femora, tibiae, and tarsi with black spots or rings; femora broad, median marking black band; midleg with one clear patellar–tibial suture and hindleg with two ones on the tibiae (Fig. 14A, indicated by red arrow). Length of femur: tibia: tarsus of foreleg = 1.8: 1.0: 1.3, femur: tibia: tarsus of midleg = 1.9: 1.0: 1.3, and femur: tibia: tarsus of hindleg = 1.8: 1.0: 1.2. Claws simple, without teeth (Fig. 14A). Abdomen with each tergite with one pair of parallel stripes near median line and tracheae with distinct pigmentation (Fig. 12A), posterolateral spines present on terga II–IX, surface with short, thick, sparse setae (Fig. 15A). Abdominal sternum (Fig. 14C) light yellow with dark spots and oblique dashes similar to that of winged stages; surface with short thick sparse setae (Fig. 14D); posterior margin of sternum IX of male and female concave (Fig. 15B, C). Middle instars light yellow without pigmentation (Fig. 14B). Caudal filaments with dark bands at the top of each segment, each segment with whorls of short, thick setae apically and long, hair-like setae laterally (Fig. 15D). Gills double on segments I and II; dorsal lamella of gill I (Fig. 16A) triangle, small, anterior rim relatively short, posterior margin straight. Dorsal lamella of gill II (Fig. 16C) leaf-shaped, anterior rim relatively short, posterior margin round, apically pointed. Ventral lamellae of gills I (Fig. 16B) and II (Fig. 16D) heart-shaped and their posterior margin slightly notched. Gills III (Fig. 5E), single, leaf-shaped, anterior rim relatively long, posterior margin round, apically slightly pointed. Gills IV–VII (Fig. 16F–J) single, oval, apically round, anterior rim relatively long; posterior margin round. Anterior rims of each gill with very short stout setae (Fig. 16J). Gill size gradually increased from gill I to gill III and decreasing from gill III to gill VII.

#### Etymology.

The specific epithet *dongxi*, is named after the Chinese abbreviation of the Institute of Eastern-Himalaya Biodiversity Research, Dali University (https://www.eastern-himalaya.com.cn/contents/16/923.html). We hope that the Institute of Eastern-Himalaya Biodiversity Research can become an important platform for biodiversity research in the world. At the same time, we hope to cooperate with scientists from all countries through the International Centre of Biodiversity and Primate Conservation (http://www.icbpc.org/index.html).

#### Distribution.

China (Yunnan).

#### Ecology.

**Figure 17.**
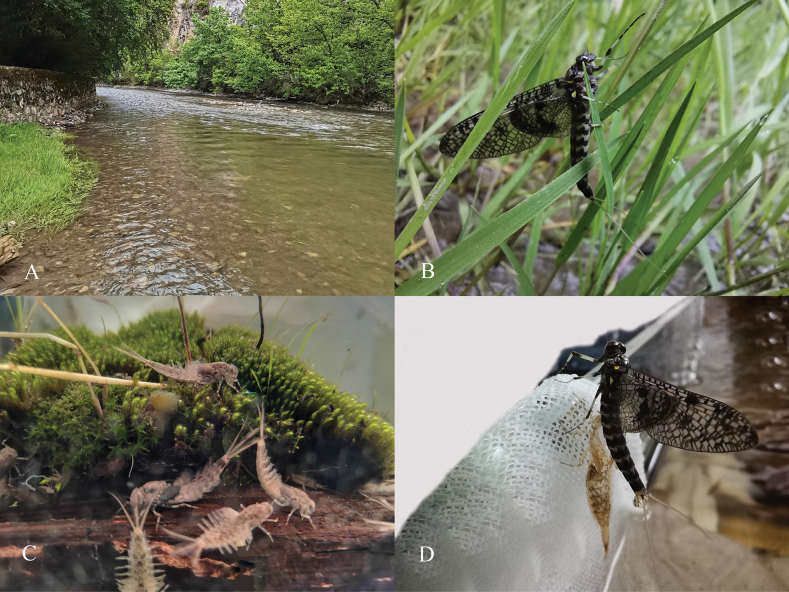
Habitat of *Siphlonurusdongxi* Li & Tong, sp. nov. **A** nymphal habitat in Dugang River **B** female subimago in the field (living) **C** nymphal habitat in laboratory (living) **D** male subimago in laboratory (living).

Nymphs of this new species prefer to live in pools or slow current areas with aquatic plants close to the bank in clear, high-altitude, wide streams (Fig. 17A). The last instar nymphs molted at noon and stayed on the grass for a relatively long time (Fig. 17B). In the laboratory, the larvae showed a behaviour of tearing water grass and collecting sediment (Fig. 17C). The emergence time is consistent with that observed in the field, the mature larvae crawled out of the water, the subimago stopped for a short time to drain a little water from the end of the abdomen before taking off (Fig. 17D). The subimago stage persisted until the third night while the observed lifespan of imagoes was about 4 days.

## ﻿Discussion

The identification key of three Asian *Siphlonurus* species, *S.binotatus* Eaton, 1892 (Gose 1979: fig. 59), *S.davidi* (Han et al. 2016: figs 3–6), *S.palaearcticus* (Tshernova, 1930) (Kluge 1982: fig. 3) with coloured wings was provided by Sartori and Peters (2004) and Han et al. (2016). However, *S.yoshinoensis* Gose, 1979, with colourful wings (Takayanagi 2021: figs 1–3), was ignored. In Asia, five species with colourful wings have been identified. Among them, the imagoes of *S.binotatus* and *S.palaearcticus* have ventral penis lobe with teeth, which can be differentiated from the other three species; *S.davidi* and *S.dongxi* Li & Tong, sp. nov. feature a fused penis lobe, while *S.yoshinoensis* has a penis lobe with a deep median incision.

*Siphlonurusdongxi* Li & Tong, sp. nov. is closely related to *S.davidi*, whose adults share the markings of the wings, the strong curvature of vein CuP, the broad expansion of the hind wing, the longer cubital area in the forewing, and the membranous penis lobes fused without teeth. *Siphlonurusdongxi* Li & Tong, sp. nov. and *S.davidi* differ from all other described *Siphlonurus* species in these characters (Sartori and Peters 2004; Han et al. 2016), which offer support that a new species complex, the *Siphlonurusdavidi* group, should be distinguished.

There are significant differences within the *Siphlonurusdavidi* group, such as the following characters:

1. Imagoes of *S.davidi* are reddish brown, while *S.dongxi* Li & Tong, sp. nov. are light yellow and dark brown.
2. Forking point of MP in forewing of *S.davidi* is subequal to the fusion point of MA and RS, then MP _2_ bends backwards strongly near to CuA. This condition is common in Ephemeridae and Potamanthidae, and is similar to *Siphlonuruschinensis* (Ulmer, 1920; Han et al. 2016), but it is not found in *S.dongxi* Li & Tong, sp. nov..
3. Penis of *S.davidi* has only membranous lobe, but the penis lobe of *S.dongxi* Li & Tong, sp. nov. has long elongations of ventral sclerite.
4. Posterolateral spines of tergum IX of *S.dongxi* Li & Tong, sp. nov. imagoes are well developed. While, the ones of *S.davidi* are poorly developed.
5. All abdominal terga of *S.davidi* nymph have distinct posterolateral spines, while the spines of *S.dongxi* Li & Tong, sp. nov. are only on segments 2–9.
6. The egg exochorionic surface of *S.dongxi* Li & Tong, sp. nov. has micropyle, but in *S.davidi* the egg exochorionic surface is without micropyle (Han et al. 2016: fig. 7).

These numerous and significant differences between *S.dongxi* Li & Tong, sp. nov. and *S.davidi* suggest that the existence of a species bridging the gap between them is possible.

Obviously, the new species shows characteristics that fall somewhere between *S.davidi* and other ones. The discovery of this new species bridges the gap between *S.davidi* and other *Siphlonurus* species, and could help reveal the origin and evolution of the genus *Siphlonurus*.

## Supplementary Material

XML Treatment for Siphlonurus dongxi

## ﻿Additional information

### Author ORCIDs

Xian-Fu Li https://orcid.org/0000-0002-1258-1573

### Data availability

All of the data that support the findings of this study are available in the main text or Supplementary Information.
